# 2,2′-{[(1*E*,1′*E*)-(Cyclo­hexane-1,4-diyl)­bis(aza­nylyl­idene)]bis­(ethan-1-yl-1-yl­idene)}diphenol

**DOI:** 10.1107/S1600536813026123

**Published:** 2013-09-28

**Authors:** S. H. Anjana Lakshmi, M. Kandaswamy, V. Ramkumar

**Affiliations:** a Department of Inorganic Chemistry, University of Madras, Maraimalai Campus (Guindy), Chennai-25, India; b Department of Chemistry, IIT Madras, Chennai 600 036, TamilNadu, India

## Abstract

The title compound, C_22_H_26_N_2_O_2_, crystallizes with three independent mol­ecules, two of which are situated on inversion centers, so the asymmetric unit contains two independent half-mol­ecules and one mol­ecule in a general position. The two hy­droxy groups in each mol­ecule are involved in intra­molecular O—H⋯N hydrogen bonds, which generate *S*(6) rings. In the crystal, weak inter­molecular C—H⋯π inter­actions link the mol­ecules into two crystallographically independent columns propagating along [001]; one column consists of mol­ecules in general positions, while the other column is built from alternating independent centrosymmetric mol­ecules.

## Related literature

For applications of Schiff base ligands in coordination chemistry, see: Gao & Zheng (2002 ▶); Hamil *et al.* (2012 ▶); Chu *et al.* (2008 ▶); More *et al.* (2001 ▶); Vigato & Tamburini (2004 ▶). For details of the synthesis, see: Huang *et al.* (2008 ▶).
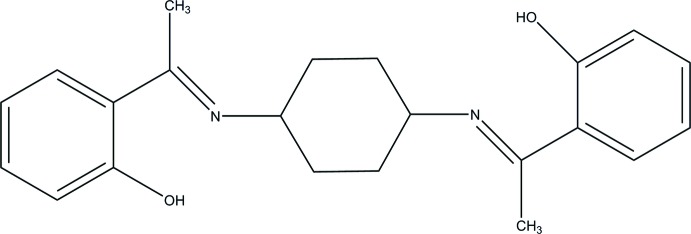


## Experimental

### 

#### Crystal data

C_22_H_26_N_2_O_2_
*M* *_r_* = 350.45
Triclinic, 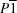

*a* = 9.0299 (6) Å
*b* = 11.4718 (8) Å
*c* = 17.9652 (13) Å
α = 92.946 (3)°
β = 95.521 (4)°
γ = 91.204 (3)°
*V* = 1849.3 (2) Å^3^
*Z* = 4
Mo *K*α radiation
μ = 0.08 mm^−1^
*T* = 298 K
0.25 × 0.20 × 0.10 mm

#### Data collection

Bruker APEXII CCD area-detector diffractometer
Absorption correction: multi-scan (*SADABS*; Bruker, 2004 ▶) *T*_min_ = 0.980, *T*_max_ = 0.992
20050 measured reflections
6387 independent reflections
2646 reflections with *I* > 2σ(*I*)
*R*_int_ = 0.033

#### Refinement

*R*[*F* ^2^ > 2σ(*F* ^2^)] = 0.060
*wR*(*F* ^2^) = 0.239
*S* = 1.02
6387 reflections
477 parameters
H-atom parameters constrained
Δρ_max_ = 0.30 e Å^−3^
Δρ_min_ = −0.23 e Å^−3^

### 

Data collection: *APEX2* (Bruker, 2004 ▶); cell refinement: *SAINT-Plus* (Bruker, 2004 ▶); data reduction: *SAINT-Plus*; program(s) used to solve structure: *SHELXS97* (Sheldrick, 2008 ▶); program(s) used to refine structure: *SHELXL97* (Sheldrick, 2008 ▶); molecular graphics: *ORTEP-3 for Windows* (Farrugia, 2012 ▶); software used to prepare material for publication: *SHELXL97* (Sheldrick, 2008 ▶).

## Supplementary Material

Crystal structure: contains datablock(s) global, I. DOI: 10.1107/S1600536813026123/cv5426sup1.cif

Structure factors: contains datablock(s) I. DOI: 10.1107/S1600536813026123/cv5426Isup2.hkl

Additional supplementary materials:  crystallographic information; 3D view; checkCIF report

## Figures and Tables

**Table 1:** Hydrogen-bond geometry (Å, °) *Cg*1, *Cg*2, *Cg*3 and *Cg*4 are the centroiods of the C17–C22, C1–C6, C34–C39 and C23–C28 benzene rings, respectively.

*D*—H⋯*A*	*D*—H	H⋯*A*	*D*⋯*A*	*D*—H⋯*A*
O6—H6⋯N4	0.82	1.79	2.526 (5)	147
O5—H5⋯N3	0.82	1.80	2.523 (5)	147
O2—H2*A*⋯N2	0.82	1.80	2.516 (5)	145
O1—H1*A*⋯N1	0.82	1.79	2.520 (5)	147
C11—H11*A*⋯*Cg*1^i^	0.97	2.64	3.552 (3)	155
C14—H14*A*⋯*Cg*2^ii^	0.97	2.63	3.540 (1)	156
C33—H33*B*⋯*Cg*3^iii^	0.97	2.62	3.510 (4)	151
C44—H44*A*⋯*Cg*4^iv^	0.97	2.61	3.504 (4)	152

Symmetry codes: (i) 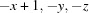; (ii) 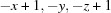; (iii) 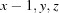; (iv) 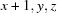.

**Fig. 1.**
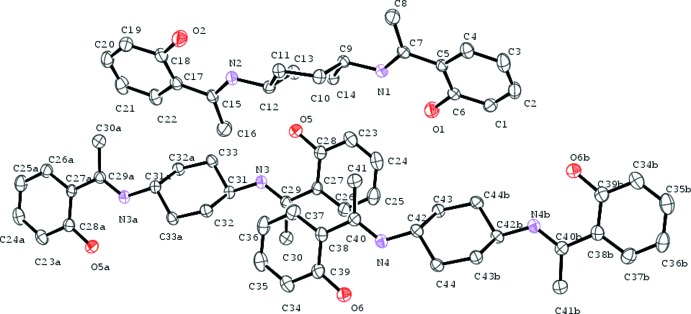
Three crystallographically independent molecules showing the atomic numbering and 30% probability displacement ellipsoids [symmetry codes: (a) -x, 1 - y, -z; (b) 2 - x, 1 - y, 1 - z]. H atoms omitted for clarity.

## supplementary crystallographic information

### 1. Comment

The research field dealing with Schiff base coordination chemistry has expanded enormously (Chu *et al.*, 2008; Gao *et al.*, 2002; Hamil *et al.*, 2012); the presence of a lone pair of electrons in an *sp*^2^ hybridized orbital of nitrogen atom of the azomethine group is of considerable chemical and biological importance (More *et al.*, 2001). Because of the relative easiness of preparation, synthetic flexibility, and the special property of C=N group, Schiff bases are generally excellent chelating agents (Vigato *et al.*, 2004). In azomethine derivatives, the C=N linkage is essential for biological activity, several azomethines were reported to possess remarkable antibacterial, antifungal, anticancer and diuretic activities. Herewith we present the crystal structure of the title compound.

The title compound crystallizes with two half-molecules situated on inversion centers and one molecule in general position (Fig. 1). Two hydroxy groups in each molecule are involved in intramolecular O—H···N hydrogen bonds (Table 1), which generate S(6) rings. In the crystal, weak intermolecular C—H···π interactions (Table 1) link the molecules into two crystallographically independent columns propagated in [001] - one column consists from the molecules in general positions, while another column is built from the alternating independent centrosymmetric molecules.

### 2. Experimental

The title compound was prepared by treating trans1, 4-diamino cyclohexane with 2-hydroxy acetophenone in the stoichiometric ratio in the ethanolic solution and refluxed for 5 h. The reaction mixture was then cooled slowly to room temperature; a yellow crystalline product was obtained and further washed with cold ethanol respectively (Huang *et al.*, 2008). It was then recrystallized from chloroform. The yellow like single-crystal of the title compound used in X-ray diffraction studies were grown in a chloroform solution by slow evaporation of the solvent at room temperature.

### 3. Refinement

All hydrogen atoms were fixed geometrically (C—H 0.93–0.98 Å) and refined as riding, with *U*_iso_(H) = 1.2–1.5 *U*_eq_(C).

### Crystal data

**Table:**

C_22_H_26_N_2_O_2_	*Z* = 4
*M**_r_* = 350.45	*F*(000) = 752
Triclinic, *P*1	*D*_x_ = 1.259 Mg m^−^^3^
Hall symbol: -P 1	Mo *K*α radiation, λ = 0.71073 Å
*a* = 9.0299 (6) Å	Cell parameters from 5628 reflections
*b* = 11.4718 (8) Å	θ = 0.0–0.0°
*c* = 17.9652 (13) Å	µ = 0.08 mm^−^^1^
α = 92.946 (3)°	*T* = 298 K
β = 95.521 (4)°	Block, yellow
γ = 91.204 (3)°	0.25 × 0.20 × 0.10 mm
*V* = 1849.3 (2) Å^3^	

### Data collection

**Table:**

Bruker APEXII CCD area-detector diffractometer	6387 independent reflections
Radiation source: fine-focus sealed tube	2646 reflections with *I* > 2σ(*I*)
Graphite monochromator	*R*_int_ = 0.033
phi and ω scans	θ_max_ = 25.0°, θ_min_ = 1.1°
Absorption correction: multi-scan (*SADABS*; Bruker, 2004)	*h* = −10→10
*T*_min_ = 0.980, *T*_max_ = 0.992	*k* = −13→13
20050 measured reflections	*l* = −21→21

### Refinement

**Table:**

Refinement on *F*^2^	Primary atom site location: structure-invariant direct methods
Least-squares matrix: full	Secondary atom site location: difference Fourier map
*R*[*F*^2^ > 2σ(*F*^2^)] = 0.060	Hydrogen site location: inferred from neighbouring sites
*wR*(*F*^2^) = 0.239	H-atom parameters constrained
*S* = 1.02	*w* = 1/[σ^2^(*F*_o_^2^) + (0.0873*P*)^2^ + 2.2422*P*] where *P* = (*F*_o_^2^ + 2*F*_c_^2^)/3
6387 reflections	(Δ/σ)_max_ = 0.005
477 parameters	Δρ_max_ = 0.30 e Å^−^^3^
0 restraints	Δρ_min_ = −0.23 e Å^−^^3^

### Special details

**Table:**

Geometry. All e.s.d.'s (except the e.s.d. in the dihedral angle between two l.s. planes)are estimated using the full covariance matrix. The cell e.s.d.'s are takeninto account individually in the estimation of e.s.d.'s in distances, anglesand torsion angles; correlations between e.s.d.'s in cell parameters are onlyused when they are defined by crystal symmetry. An approximate (isotropic)treatment of cell e.s.d.'s is used for estimating e.s.d.'s involving l.s. planes.
Refinement. Refinement of *F*^2^ against ALL reflections. The weighted *R*-factor *wR* andgoodness of fit *S* are based on *F*^2^, conventional *R*-factors *R* are basedon *F*, with *F* set to zero for negative *F*^2^. The threshold expression of*F*^2^ > σ(*F*^2^) is used only for calculating *R*-factors(gt) *etc*. and isnot relevant to the choice of reflections for refinement. *R*-factors basedon *F*^2^ are statistically about twice as large as those based on *F*, and *R*-factors based on ALL data will be even larger.

### Fractional atomic coordinates and isotropic or equivalent isotropic displacement parameters (Å^2^)

**Table:**

	*x*	*y*	*z*	*U*_iso_*/*U*_eq_	
C1	0.8319 (7)	1.1440 (5)	0.6061 (3)	0.0575 (17)	
H1	0.8150	1.2200	0.6233	0.069*	
C2	0.9279 (7)	1.0768 (6)	0.6478 (3)	0.0603 (18)	
H2	0.9765	1.1078	0.6927	0.072*	
C3	0.9532 (6)	0.9640 (6)	0.6240 (3)	0.0594 (17)	
H3	1.0188	0.9187	0.6525	0.071*	
C4	0.8809 (6)	0.9185 (5)	0.5577 (3)	0.0464 (14)	
H4	0.8989	0.8422	0.5416	0.056*	
C5	0.7805 (6)	0.9842 (4)	0.5136 (3)	0.0362 (13)	
C6	0.7589 (6)	1.1000 (4)	0.5381 (3)	0.0439 (14)	
C7	0.7032 (6)	0.9339 (4)	0.4427 (3)	0.0365 (12)	
C8	0.7086 (7)	0.8049 (4)	0.4243 (3)	0.0579 (17)	
H8A	0.6480	0.7854	0.3783	0.087*	
H8B	0.6718	0.7631	0.4640	0.087*	
H8C	0.8095	0.7838	0.4189	0.087*	
C9	0.5504 (6)	0.9694 (4)	0.3276 (3)	0.0353 (13)	
H9	0.5033	0.8923	0.3307	0.042*	
C10	0.6546 (6)	0.9646 (4)	0.2661 (3)	0.0408 (13)	
H10A	0.7259	0.9037	0.2754	0.049*	
H10B	0.7095	1.0383	0.2669	0.049*	
C11	0.5697 (6)	0.9406 (4)	0.1895 (3)	0.0398 (13)	
H11A	0.6391	0.9416	0.1516	0.048*	
H11B	0.5234	0.8632	0.1873	0.048*	
C12	0.4503 (6)	1.0297 (4)	0.1724 (3)	0.0389 (13)	
H12	0.4978	1.1067	0.1694	0.047*	
C13	0.3456 (6)	1.0352 (4)	0.2340 (3)	0.0418 (13)	
H13A	0.2750	1.0966	0.2247	0.050*	
H13B	0.2899	0.9618	0.2332	0.050*	
C14	0.4303 (6)	1.0587 (4)	0.3110 (3)	0.0396 (13)	
H14A	0.3608	1.0570	0.3488	0.048*	
H14B	0.4763	1.1362	0.3136	0.048*	
C15	0.2981 (6)	1.0670 (4)	0.0578 (3)	0.0405 (13)	
C16	0.2927 (7)	1.1947 (4)	0.0751 (3)	0.0628 (18)	
H16A	0.3690	1.2176	0.1144	0.094*	
H16B	0.1971	1.2134	0.0909	0.094*	
H16C	0.3085	1.2356	0.0311	0.094*	
C17	0.2196 (6)	1.0170 (5)	−0.0128 (3)	0.0425 (14)	
C18	0.2409 (7)	0.9003 (5)	−0.0371 (3)	0.0507 (15)	
C19	0.1686 (7)	0.8566 (5)	−0.1058 (3)	0.0591 (17)	
H19	0.1866	0.7810	−0.1233	0.071*	
C20	0.0720 (7)	0.9235 (6)	−0.1473 (3)	0.0608 (18)	
H20	0.0233	0.8924	−0.1921	0.073*	
C21	0.0462 (7)	1.0362 (6)	−0.1235 (3)	0.0608 (18)	
H21	−0.0203	1.0811	−0.1517	0.073*	
C22	0.1200 (6)	1.0821 (5)	−0.0574 (3)	0.0509 (15)	
H22	0.1029	1.1588	−0.0419	0.061*	
C23	0.3019 (7)	0.3440 (5)	0.3478 (3)	0.0517 (15)	
H23	0.2733	0.2697	0.3603	0.062*	
C24	0.4097 (6)	0.4066 (5)	0.3931 (3)	0.0570 (17)	
H24	0.4552	0.3738	0.4354	0.068*	
C25	0.4508 (6)	0.5175 (5)	0.3762 (3)	0.0584 (17)	
H25	0.5239	0.5595	0.4071	0.070*	
C26	0.3841 (6)	0.5662 (5)	0.3139 (3)	0.0487 (15)	
H26	0.4127	0.6415	0.3034	0.058*	
C27	0.2739 (6)	0.5058 (4)	0.2651 (3)	0.0378 (12)	
C28	0.2345 (6)	0.3910 (4)	0.2828 (3)	0.0418 (14)	
C29	0.2037 (6)	0.5571 (4)	0.1977 (3)	0.0398 (13)	
C30	0.2285 (6)	0.6845 (4)	0.1877 (3)	0.0567 (16)	
H30A	0.1619	0.7084	0.1467	0.085*	
H30B	0.3294	0.6986	0.1773	0.085*	
H30C	0.2100	0.7284	0.2327	0.085*	
C31	0.0496 (6)	0.5264 (4)	0.0803 (3)	0.0379 (13)	
H31	0.0089	0.6039	0.0888	0.045*	
C32	0.1573 (6)	0.5315 (4)	0.0199 (3)	0.0424 (13)	
H32A	0.2344	0.5905	0.0350	0.051*	
H32B	0.2046	0.4568	0.0146	0.051*	
C33	−0.0771 (6)	0.4398 (4)	0.0546 (3)	0.0420 (13)	
H33A	−0.0377	0.3619	0.0502	0.050*	
H33B	−0.1476	0.4398	0.0921	0.050*	
C34	0.6985 (7)	0.6551 (5)	0.1526 (3)	0.0535 (16)	
H34	0.7273	0.7295	0.1404	0.064*	
C35	0.5913 (7)	0.5937 (6)	0.1067 (3)	0.0614 (18)	
H35	0.5471	0.6267	0.0641	0.074*	
C36	0.5488 (6)	0.4827 (6)	0.1238 (3)	0.0605 (18)	
H36	0.4750	0.4411	0.0932	0.073*	
C37	0.6156 (6)	0.4339 (5)	0.1860 (3)	0.0521 (15)	
H37	0.5873	0.3585	0.1964	0.063*	
C38	0.7259 (6)	0.4945 (4)	0.2347 (3)	0.0403 (13)	
C39	0.7655 (6)	0.6083 (4)	0.2172 (3)	0.0450 (14)	
C40	0.7956 (6)	0.4409 (4)	0.3026 (3)	0.0382 (13)	
C41	0.7717 (7)	0.3141 (4)	0.3121 (3)	0.0582 (18)	
H41A	0.8326	0.2915	0.3555	0.087*	
H41B	0.7982	0.2703	0.2686	0.087*	
H41C	0.6689	0.2987	0.3184	0.087*	
C42	0.9513 (5)	0.4732 (4)	0.4197 (3)	0.0382 (13)	
H42	0.9922	0.3957	0.4113	0.046*	
C43	0.8430 (5)	0.4678 (4)	0.4798 (3)	0.0398 (13)	
H43A	0.7947	0.5422	0.4846	0.048*	
H43B	0.7666	0.4083	0.4647	0.048*	
C44	1.0776 (6)	0.5597 (4)	0.4452 (3)	0.0391 (13)	
H44A	1.1486	0.5588	0.4079	0.047*	
H44B	1.0383	0.6376	0.4489	0.047*	
N1	0.6309 (5)	1.0050 (3)	0.3999 (2)	0.0394 (11)	
N2	0.3692 (5)	0.9952 (3)	0.1003 (2)	0.0408 (11)	
N3	0.1238 (5)	0.4889 (3)	0.1507 (2)	0.0397 (11)	
N4	0.8768 (5)	0.5101 (3)	0.3495 (2)	0.0406 (11)	
O1	0.6695 (6)	1.1708 (3)	0.4976 (2)	0.0688 (13)	
H1A	0.6349	1.1355	0.4588	0.103*	
O2	0.3297 (6)	0.8290 (3)	0.0025 (2)	0.0714 (13)	
H2A	0.3707	0.8653	0.0397	0.107*	
O5	0.1332 (5)	0.3254 (3)	0.2392 (2)	0.0574 (11)	
H5	0.1070	0.3598	0.2014	0.086*	
O6	0.8666 (5)	0.6742 (3)	0.2608 (2)	0.0581 (11)	
H6	0.8922	0.6402	0.2987	0.087*	

### Atomic displacement parameters (Å^2^)

**Table:**

	*U*^11^	*U*^22^	*U*^33^	*U*^12^	*U*^13^	*U*^23^
C1	0.079 (5)	0.054 (3)	0.039 (3)	−0.023 (3)	0.012 (3)	−0.002 (3)
C2	0.056 (4)	0.088 (5)	0.035 (3)	−0.022 (3)	−0.002 (3)	0.007 (3)
C3	0.044 (4)	0.093 (5)	0.041 (4)	−0.001 (3)	−0.002 (3)	0.012 (3)
C4	0.040 (4)	0.060 (3)	0.041 (3)	0.004 (3)	0.007 (3)	0.007 (3)
C5	0.036 (3)	0.042 (3)	0.031 (3)	−0.005 (2)	0.004 (3)	0.006 (2)
C6	0.057 (4)	0.041 (3)	0.035 (3)	−0.007 (3)	0.008 (3)	0.006 (2)
C7	0.035 (3)	0.040 (3)	0.036 (3)	0.002 (2)	0.010 (3)	0.007 (2)
C8	0.077 (5)	0.044 (3)	0.051 (4)	0.012 (3)	−0.007 (3)	0.004 (3)
C9	0.036 (3)	0.038 (3)	0.032 (3)	−0.002 (2)	0.004 (3)	0.004 (2)
C10	0.031 (3)	0.049 (3)	0.042 (3)	0.003 (2)	0.001 (3)	0.001 (2)
C11	0.035 (3)	0.048 (3)	0.037 (3)	0.003 (2)	0.004 (3)	0.002 (2)
C12	0.039 (3)	0.039 (3)	0.039 (3)	0.001 (2)	0.001 (3)	0.001 (2)
C13	0.036 (3)	0.051 (3)	0.039 (3)	0.004 (2)	0.006 (3)	0.003 (2)
C14	0.038 (4)	0.047 (3)	0.035 (3)	0.004 (2)	0.009 (3)	0.001 (2)
C15	0.040 (3)	0.049 (3)	0.032 (3)	−0.001 (2)	0.005 (3)	0.006 (3)
C16	0.077 (5)	0.051 (3)	0.058 (4)	0.006 (3)	−0.009 (4)	0.003 (3)
C17	0.039 (4)	0.055 (3)	0.036 (3)	−0.004 (3)	0.011 (3)	0.007 (3)
C18	0.055 (4)	0.056 (4)	0.040 (3)	−0.008 (3)	−0.002 (3)	0.009 (3)
C19	0.070 (5)	0.061 (4)	0.045 (4)	−0.017 (3)	0.007 (4)	−0.002 (3)
C20	0.053 (5)	0.087 (5)	0.040 (4)	−0.021 (3)	0.000 (3)	−0.001 (3)
C21	0.037 (4)	0.097 (5)	0.048 (4)	0.001 (3)	−0.005 (3)	0.014 (3)
C22	0.042 (4)	0.069 (4)	0.042 (3)	0.006 (3)	0.002 (3)	0.009 (3)
C23	0.061 (4)	0.053 (3)	0.044 (3)	0.018 (3)	0.015 (3)	0.006 (3)
C24	0.051 (4)	0.083 (4)	0.038 (3)	0.019 (3)	0.004 (3)	0.007 (3)
C25	0.040 (4)	0.087 (4)	0.046 (4)	0.000 (3)	0.000 (3)	−0.005 (3)
C26	0.043 (4)	0.062 (4)	0.041 (3)	−0.007 (3)	0.008 (3)	−0.002 (3)
C27	0.038 (3)	0.045 (3)	0.030 (3)	0.003 (2)	0.008 (3)	−0.005 (2)
C28	0.040 (4)	0.047 (3)	0.039 (3)	0.005 (3)	0.010 (3)	−0.002 (3)
C29	0.041 (3)	0.043 (3)	0.037 (3)	0.001 (2)	0.007 (3)	0.002 (2)
C30	0.069 (4)	0.051 (3)	0.049 (3)	−0.006 (3)	0.004 (3)	0.001 (3)
C31	0.038 (4)	0.042 (3)	0.033 (3)	0.005 (2)	0.002 (3)	0.001 (2)
C32	0.034 (3)	0.052 (3)	0.042 (3)	−0.001 (2)	0.005 (3)	0.005 (2)
C33	0.039 (3)	0.053 (3)	0.035 (3)	0.000 (2)	0.007 (3)	0.008 (2)
C34	0.059 (4)	0.058 (3)	0.045 (3)	0.016 (3)	0.009 (3)	0.009 (3)
C35	0.050 (4)	0.089 (5)	0.046 (4)	0.023 (4)	0.002 (3)	0.005 (3)
C36	0.040 (4)	0.092 (5)	0.047 (4)	0.007 (3)	−0.008 (3)	−0.006 (3)
C37	0.039 (4)	0.064 (4)	0.053 (4)	−0.005 (3)	0.007 (3)	−0.004 (3)
C38	0.035 (4)	0.052 (3)	0.035 (3)	0.006 (3)	0.011 (3)	−0.001 (2)
C39	0.052 (4)	0.043 (3)	0.041 (3)	0.009 (3)	0.009 (3)	−0.005 (3)
C40	0.035 (4)	0.045 (3)	0.036 (3)	0.001 (2)	0.014 (3)	−0.006 (2)
C41	0.074 (5)	0.046 (3)	0.052 (4)	−0.007 (3)	−0.004 (4)	0.004 (3)
C42	0.039 (3)	0.039 (3)	0.038 (3)	0.005 (2)	0.006 (3)	0.006 (2)
C43	0.032 (3)	0.049 (3)	0.039 (3)	0.000 (2)	0.003 (3)	0.005 (2)
C44	0.035 (3)	0.045 (3)	0.039 (3)	0.001 (2)	0.011 (3)	0.007 (2)
N1	0.041 (3)	0.040 (2)	0.037 (2)	−0.0011 (19)	0.002 (2)	0.0043 (19)
N2	0.041 (3)	0.048 (3)	0.034 (3)	0.001 (2)	0.002 (2)	0.003 (2)
N3	0.040 (3)	0.046 (2)	0.034 (2)	0.002 (2)	0.004 (2)	0.0005 (19)
N4	0.044 (3)	0.044 (2)	0.035 (2)	0.002 (2)	0.005 (2)	0.0022 (19)
O1	0.111 (4)	0.040 (2)	0.052 (3)	0.008 (2)	−0.008 (3)	−0.0017 (19)
O2	0.104 (4)	0.046 (2)	0.059 (3)	0.008 (2)	−0.015 (3)	0.000 (2)
O5	0.077 (3)	0.044 (2)	0.048 (2)	−0.008 (2)	−0.005 (2)	0.0011 (18)
O6	0.079 (3)	0.043 (2)	0.050 (3)	−0.006 (2)	−0.004 (2)	0.0034 (18)

### Geometric parameters (Å, º)

**Table:**

C1—C2	1.367 (8)	C24—C25	1.374 (7)
C1—C6	1.397 (7)	C24—H24	0.9300
C1—H1	0.9300	C25—C26	1.370 (7)
C2—C3	1.373 (8)	C25—H25	0.9300
C2—H2	0.9300	C26—C27	1.407 (6)
C3—C4	1.375 (7)	C26—H26	0.9300
C3—H3	0.9300	C27—C28	1.417 (7)
C4—C5	1.403 (6)	C27—C29	1.468 (7)
C4—H4	0.9300	C28—O5	1.338 (5)
C5—C6	1.402 (6)	C29—N3	1.279 (6)
C5—C7	1.476 (6)	C29—C30	1.497 (6)
C6—O1	1.346 (6)	C30—H30A	0.9600
C7—N1	1.293 (6)	C30—H30B	0.9600
C7—C8	1.502 (6)	C30—H30C	0.9600
C8—H8A	0.9600	C31—N3	1.462 (6)
C8—H8B	0.9600	C31—C33	1.522 (6)
C8—H8C	0.9600	C31—C32	1.528 (7)
C9—N1	1.460 (5)	C31—H31	0.9800
C9—C10	1.519 (7)	C32—C33^i^	1.514 (6)
C9—C14	1.526 (6)	C32—H32A	0.9700
C9—H9	0.9800	C32—H32B	0.9700
C10—C11	1.519 (6)	C33—C32^i^	1.514 (6)
C10—H10A	0.9700	C33—H33A	0.9700
C10—H10B	0.9700	C33—H33B	0.9700
C11—C12	1.522 (6)	C34—C35	1.367 (7)
C11—H11A	0.9700	C34—C39	1.394 (7)
C11—H11B	0.9700	C34—H34	0.9300
C12—N2	1.457 (6)	C35—C36	1.381 (8)
C12—C13	1.524 (7)	C35—H35	0.9300
C12—H12	0.9800	C36—C37	1.368 (7)
C13—C14	1.523 (6)	C36—H36	0.9300
C13—H13A	0.9700	C37—C38	1.407 (7)
C13—H13B	0.9700	C37—H37	0.9300
C14—H14A	0.9700	C38—C39	1.406 (7)
C14—H14B	0.9700	C38—C40	1.485 (7)
C15—N2	1.291 (6)	C39—O6	1.338 (6)
C15—C17	1.474 (7)	C40—N4	1.290 (6)
C15—C16	1.484 (7)	C40—C41	1.487 (6)
C16—H16A	0.9600	C41—H41A	0.9600
C16—H16B	0.9600	C41—H41B	0.9600
C16—H16C	0.9600	C41—H41C	0.9600
C17—C22	1.400 (7)	C42—N4	1.459 (6)
C17—C18	1.409 (7)	C42—C44	1.517 (6)
C18—O2	1.341 (6)	C42—C43	1.528 (6)
C18—C19	1.403 (7)	C42—H42	0.9800
C19—C20	1.367 (8)	C43—C44^ii^	1.516 (6)
C19—H19	0.9300	C43—H43A	0.9700
C20—C21	1.372 (8)	C43—H43B	0.9700
C20—H20	0.9300	C44—C43^ii^	1.516 (6)
C21—C22	1.379 (7)	C44—H44A	0.9700
C21—H21	0.9300	C44—H44B	0.9700
C22—H22	0.9300	O1—H1A	0.8200
C23—C24	1.373 (7)	O2—H2A	0.8200
C23—C28	1.402 (7)	O5—H5	0.8201
C23—H23	0.9300	O6—H6	0.8201
			
C2—C1—C6	120.8 (6)	C23—C24—H24	119.6
C2—C1—H1	119.4	C25—C24—H24	120.0
C6—C1—H1	119.8	C26—C25—C24	120.0 (5)
C3—C2—C1	120.6 (5)	C26—C25—H25	120.0
C3—C2—H2	119.7	C24—C25—H25	120.0
C1—C2—H2	119.7	C25—C26—C27	122.1 (5)
C2—C3—C4	119.5 (5)	C25—C26—H26	119.0
C2—C3—H3	120.4	C27—C26—H26	118.9
C4—C3—H3	120.1	C26—C27—C28	117.1 (5)
C3—C4—C5	121.7 (5)	C26—C27—C29	122.2 (5)
C3—C4—H4	119.2	C28—C27—C29	120.6 (4)
C5—C4—H4	119.1	O5—C28—C23	118.5 (5)
C4—C5—C6	117.9 (4)	O5—C28—C27	121.8 (5)
C4—C5—C7	121.1 (4)	C23—C28—C27	119.8 (5)
C6—C5—C7	121.0 (4)	N3—C29—C27	117.3 (4)
O1—C6—C1	118.7 (5)	N3—C29—C30	123.8 (5)
O1—C6—C5	121.8 (4)	C27—C29—C30	118.9 (4)
C1—C6—C5	119.5 (5)	C29—C30—H30A	109.5
N1—C7—C5	117.1 (4)	C29—C30—H30B	109.7
N1—C7—C8	123.3 (4)	H30A—C30—H30B	109.5
C5—C7—C8	119.6 (4)	C29—C30—H30C	109.2
C7—C8—H8A	109.6	H30A—C30—H30C	109.5
C7—C8—H8B	109.5	H30B—C30—H30C	109.5
H8A—C8—H8B	109.5	N3—C31—C33	108.2 (4)
C7—C8—H8C	109.4	N3—C31—C32	111.4 (4)
H8A—C8—H8C	109.5	C33—C31—C32	109.6 (4)
H8B—C8—H8C	109.5	N3—C31—H31	109.3
N1—C9—C10	110.9 (4)	C33—C31—H31	109.1
N1—C9—C14	107.6 (4)	C32—C31—H31	109.2
C10—C9—C14	110.1 (4)	C33^i^—C32—C31	111.1 (4)
N1—C9—H9	109.4	C33^i^—C32—H32A	109.4
C10—C9—H9	109.4	C31—C32—H32A	109.3
C14—C9—H9	109.3	C33^i^—C32—H32B	109.6
C9—C10—C11	111.4 (4)	C31—C32—H32B	109.4
C9—C10—H10A	109.2	H32A—C32—H32B	108.0
C11—C10—H10A	109.4	C32^i^—C33—C31	112.3 (4)
C9—C10—H10B	109.6	C32^i^—C33—H33A	109.3
C11—C10—H10B	109.3	C31—C33—H33A	109.0
H10A—C10—H10B	107.9	C32^i^—C33—H33B	109.1
C12—C11—C10	112.4 (4)	C31—C33—H33B	109.2
C12—C11—H11A	109.1	H33A—C33—H33B	107.9
C10—C11—H11A	109.1	C35—C34—C39	121.3 (5)
C12—C11—H11B	109.1	C35—C34—H34	119.3
C10—C11—H11B	109.2	C39—C34—H34	119.4
H11A—C11—H11B	107.9	C34—C35—C36	119.8 (5)
N2—C12—C11	108.3 (4)	C34—C35—H35	120.3
N2—C12—C13	110.7 (4)	C36—C35—H35	119.9
C11—C12—C13	110.0 (4)	C37—C36—C35	119.9 (5)
N2—C12—H12	109.2	C37—C36—H36	120.0
C11—C12—H12	109.3	C35—C36—H36	120.1
C13—C12—H12	109.2	C36—C37—C38	122.0 (5)
C14—C13—C12	111.6 (4)	C36—C37—H37	119.0
C14—C13—H13A	109.3	C38—C37—H37	119.1
C12—C13—H13A	109.5	C39—C38—C37	117.3 (5)
C14—C13—H13B	109.3	C39—C38—C40	121.4 (4)
C12—C13—H13B	109.1	C37—C38—C40	121.3 (5)
H13A—C13—H13B	108.0	O6—C39—C34	118.3 (5)
C13—C14—C9	112.3 (4)	O6—C39—C38	122.0 (5)
C13—C14—H14A	109.2	C34—C39—C38	119.7 (5)
C9—C14—H14A	109.2	N4—C40—C38	116.0 (4)
C13—C14—H14B	109.0	N4—C40—C41	124.3 (5)
C9—C14—H14B	109.2	C38—C40—C41	119.7 (4)
H14A—C14—H14B	107.8	C40—C41—H41A	109.4
N2—C15—C17	116.7 (4)	C40—C41—H41B	109.8
N2—C15—C16	124.4 (5)	H41A—C41—H41B	109.5
C17—C15—C16	118.9 (4)	C40—C41—H41C	109.2
C15—C16—H16A	109.4	H41A—C41—H41C	109.5
C15—C16—H16B	109.5	H41B—C41—H41C	109.5
H16A—C16—H16B	109.5	N4—C42—C44	108.4 (4)
C15—C16—H16C	109.5	N4—C42—C43	111.0 (4)
H16A—C16—H16C	109.5	C44—C42—C43	109.9 (4)
H16B—C16—H16C	109.5	N4—C42—H42	109.1
C22—C17—C18	117.5 (5)	C44—C42—H42	109.2
C22—C17—C15	121.7 (5)	C43—C42—H42	109.2
C18—C17—C15	120.7 (5)	C44^ii^—C43—C42	111.3 (4)
O2—C18—C19	118.2 (5)	C44^ii^—C43—H43A	109.2
O2—C18—C17	122.5 (5)	C42—C43—H43A	109.4
C19—C18—C17	119.3 (5)	C44^ii^—C43—H43B	109.4
C20—C19—C18	120.9 (6)	C42—C43—H43B	109.4
C20—C19—H19	119.8	H43A—C43—H43B	108.0
C18—C19—H19	119.4	C43^ii^—C44—C42	112.3 (4)
C19—C20—C21	120.7 (5)	C43^ii^—C44—H44A	109.2
C19—C20—H20	119.7	C42—C44—H44A	109.1
C21—C20—H20	119.6	C43^ii^—C44—H44B	109.0
C20—C21—C22	119.3 (6)	C42—C44—H44B	109.3
C20—C21—H21	120.2	H44A—C44—H44B	107.9
C22—C21—H21	120.5	C7—N1—C9	123.8 (4)
C21—C22—C17	122.2 (6)	C15—N2—C12	123.8 (4)
C21—C22—H22	118.8	C29—N3—C31	123.6 (4)
C17—C22—H22	119.0	C40—N4—C42	123.5 (4)
C24—C23—C28	120.6 (5)	C6—O1—H1A	109.4
C24—C23—H23	119.8	C18—O2—H2A	109.6
C28—C23—H23	119.6	C28—O5—H5	109.4
C23—C24—C25	120.4 (5)	C39—O6—H6	109.5
			
C6—C1—C2—C3	−0.8 (9)	C26—C27—C28—O5	−178.5 (5)
C1—C2—C3—C4	−0.1 (9)	C29—C27—C28—O5	0.1 (8)
C2—C3—C4—C5	−0.4 (9)	C26—C27—C28—C23	1.9 (8)
C3—C4—C5—C6	1.9 (8)	C29—C27—C28—C23	−179.5 (5)
C3—C4—C5—C7	−179.7 (5)	C26—C27—C29—N3	168.5 (5)
C2—C1—C6—O1	−177.6 (6)	C28—C27—C29—N3	−10.1 (8)
C2—C1—C6—C5	2.2 (9)	C26—C27—C29—C30	−11.7 (8)
C4—C5—C6—O1	177.1 (5)	C28—C27—C29—C30	169.7 (5)
C7—C5—C6—O1	−1.3 (8)	N3—C31—C32—C33^i^	175.1 (4)
C4—C5—C6—C1	−2.7 (8)	C33—C31—C32—C33^i^	55.4 (6)
C7—C5—C6—C1	178.9 (5)	N3—C31—C33—C32^i^	−177.7 (4)
C4—C5—C7—N1	−169.6 (5)	C32—C31—C33—C32^i^	−56.0 (6)
C6—C5—C7—N1	8.8 (7)	C39—C34—C35—C36	−0.7 (9)
C4—C5—C7—C8	11.7 (8)	C34—C35—C36—C37	−0.9 (9)
C6—C5—C7—C8	−169.9 (5)	C35—C36—C37—C38	1.2 (9)
N1—C9—C10—C11	−173.8 (4)	C36—C37—C38—C39	0.1 (8)
C14—C9—C10—C11	−54.9 (5)	C36—C37—C38—C40	179.1 (6)
C9—C10—C11—C12	56.3 (5)	C35—C34—C39—O6	−178.1 (5)
C10—C11—C12—N2	−176.4 (4)	C35—C34—C39—C38	2.0 (8)
C10—C11—C12—C13	−55.2 (5)	C37—C38—C39—O6	178.4 (5)
N2—C12—C13—C14	174.1 (4)	C40—C38—C39—O6	−0.6 (8)
C11—C12—C13—C14	54.3 (5)	C37—C38—C39—C34	−1.6 (8)
C12—C13—C14—C9	−55.5 (5)	C40—C38—C39—C34	179.4 (5)
N1—C9—C14—C13	176.0 (4)	C39—C38—C40—N4	9.9 (8)
C10—C9—C14—C13	55.0 (5)	C37—C38—C40—N4	−169.1 (5)
N2—C15—C17—C22	169.6 (5)	C39—C38—C40—C41	−169.0 (5)
C16—C15—C17—C22	−11.2 (8)	C37—C38—C40—C41	12.0 (8)
N2—C15—C17—C18	−9.4 (8)	N4—C42—C43—C44^ii^	−174.8 (4)
C16—C15—C17—C18	169.9 (5)	C44—C42—C43—C44^ii^	−54.9 (6)
C22—C17—C18—O2	−177.6 (5)	N4—C42—C44—C43^ii^	176.9 (4)
C15—C17—C18—O2	1.4 (9)	C43—C42—C44—C43^ii^	55.4 (6)
C22—C17—C18—C19	3.2 (8)	C5—C7—N1—C9	179.2 (4)
C15—C17—C18—C19	−177.8 (5)	C8—C7—N1—C9	−2.2 (8)
O2—C18—C19—C20	177.3 (6)	C10—C9—N1—C7	−83.5 (6)
C17—C18—C19—C20	−3.4 (9)	C14—C9—N1—C7	156.0 (5)
C18—C19—C20—C21	1.5 (10)	C17—C15—N2—C12	−178.9 (5)
C19—C20—C21—C22	0.6 (9)	C16—C15—N2—C12	1.8 (9)
C20—C21—C22—C17	−0.8 (9)	C11—C12—N2—C15	−155.7 (5)
C18—C17—C22—C21	−1.1 (9)	C13—C12—N2—C15	83.6 (6)
C15—C17—C22—C21	179.9 (5)	C27—C29—N3—C31	−178.6 (5)
C28—C23—C24—C25	1.4 (8)	C30—C29—N3—C31	1.6 (8)
C23—C24—C25—C26	0.0 (9)	C33—C31—N3—C29	−158.9 (5)
C24—C25—C26—C27	−0.5 (9)	C32—C31—N3—C29	80.6 (6)
C25—C26—C27—C28	−0.5 (8)	C38—C40—N4—C42	178.8 (5)
C25—C26—C27—C29	−179.1 (5)	C41—C40—N4—C42	−2.4 (8)
C24—C23—C28—O5	178.0 (5)	C44—C42—N4—C40	159.2 (5)
C24—C23—C28—C27	−2.4 (8)	C43—C42—N4—C40	−80.0 (6)

Symmetry codes: (i) −*x*, −*y*+1, −*z*; (ii) −*x*+2, −*y*+1, −*z*+1.

### Hydrogen-bond geometry (Å, º)

Cg1, Cg2, Cg3 and Cg4 are the centroiods of the C17–C22, C1–C6, C34–C39 and C23–C28 benzene rings, respectively.

**Table:**

*D*—H···*A*	*D*—H	H···*A*	*D*···*A*	*D*—H···*A*
O6—H6···N4	0.82	1.79	2.526 (5)	147
O5—H5···N3	0.82	1.80	2.523 (5)	147
O2—H2*A*···N2	0.82	1.80	2.516 (5)	145
O1—H1*A*···N1	0.82	1.79	2.520 (5)	147
C11—H11*A*···*Cg*1^iii^	0.97	2.64	3.552 (3)	155
C14—H14*A*···*Cg*2^iv^	0.97	2.63	3.540 (1)	156
C33—H33*B*···*Cg*3^v^	0.97	2.62	3.510 (4)	151
C44—H44*A*···*Cg*4^vi^	0.97	2.61	3.504 (4)	152

Symmetry codes: (iii) −*x*+1, −*y*, −*z*; (iv) −*x*+1, −*y*, −*z*+1; (v) *x*−1, *y*, *z*; (vi) *x*+1, *y*, *z*.
